# L'actinomycose cervico-faciale: à propos d'un cas

**DOI:** 10.11604/pamj.2013.14.147.2283

**Published:** 2013-04-14

**Authors:** Bouchra Badre, Mustapha Essaadi, Samira El Arabi

**Affiliations:** 1 Service de Pédodontie-Prévention-Faculté de Médecine dentaire de Casablanca, Maroc; 2 Service d'Oto-Rhino-Laryngologie-Hôpital 20 août Casablanca, Maroc

**Keywords:** Actinomycose, cervico-faciale, Actinomyces Israelii, infection, actinomycosis, cervicofacial, Actinomyces Israelii, infection

## Abstract

L'actinomycose est une infection spécifique rare, non contagieuse. Elle se localise dans la région des maxillaires, avec une évolution chronique. Le germe le plus souvent incriminé est *Maroc*. Nous rapportons le cas d'un enfant âgé de 5 ans, qui a présenté des processus suppuratifs chroniques en rapport avec une actinomycose sous mandibulaire gauche. Le diagnostic de certitude a été porté par l'examen histopathologique. Le traitement de cette infection est chirurgical avec drainage et curetage des lésions, associé à une antibiothérapie à forte dose et à durée prolongée. L'actinomycose peut simuler n'importe quelle affection de la sphère ORL, elle peut prendre en particulier un aspect pseudotumoral égarant de ce fait son diagnostic. Une sensibilisation des médecins à cette pathologie s'impose, pour un diagnostic précoce dont dépend le pronostic.

## Introduction

L'actinomycose est une infection granulomateuse chronique, suppurative, caractérisée dans les formes typiques par la formation de multiples abcès. Le germe responsable de l'actinomycose est *Maroc* [1], bien que d'autres actinomycètes puissent être impliqués [2]. Ces agents bactériens font partie de la flore normale de la cavité buccale, mais dans certaines circonstances ils peuvent devenir pathogènes [3]. L'actinomycose peut toucher tous les organes du corps, y compris la région cervico- faciale [4, 5]. Généralement, l'actinomycose cervico-faciale est située dans la région sous mandibulaire ou autour de l'angle de la mandibule, la forme cervicale est la forme anatomique la plus fréquente. En revanche, l'actinomycose de la muqueuse buccale est assez rare, surtout chez l'enfant. L'aspect clinique, le diagnostic et le traitement de cette affection inhabituelle seront discutés à travers le cas clinique d'un enfant âgé de 5 ans.

## Patient et observation

L'enfant H., âgé de 5 ans, sans antécédents pathologiques particuliers a présenté en novembre 2004 une tuméfaction sous angulo-maxillaire gauche évoluant depuis 3 semaines avant sa consultation au service de pédodontie du Chu Ibn Rochd de Casablanca.

**Figure 1:**
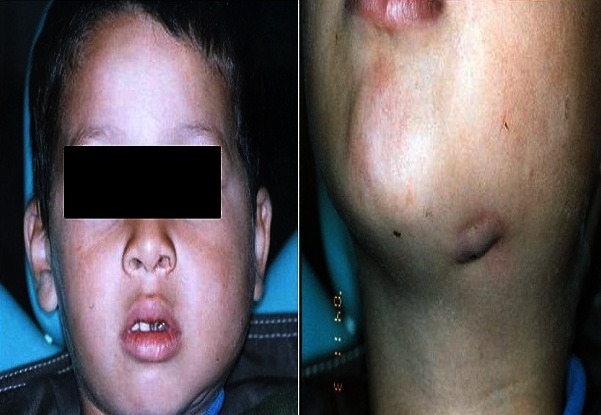
Tuméfaction sous angulaire avec adénite satellite

**Figure 2:**
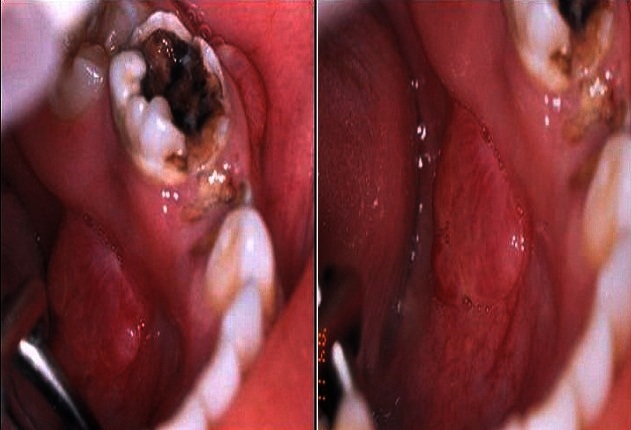
Dents très délabrées en regard de la formation tumorale

L'examen clinique retrouve un enfant apyrétique, en bon état général. L'examen local met en évidence une tuméfaction sous angulo-maxillaire gauche, ferme, non mobile, de taille moyenne et douloureuse à la palpation avec une adénopathie cervicale satellite (Figure 1). L'examen de la cavité buccale montre une formation tumorale endo-buccale fixée au maxillaire inférieur, et un mauvais état bucco-dentaire avec des molaires temporaires inférieure (74-75) très délabrées juste en regard de la formation tumorale (Figure 2).

**Figure 3:**
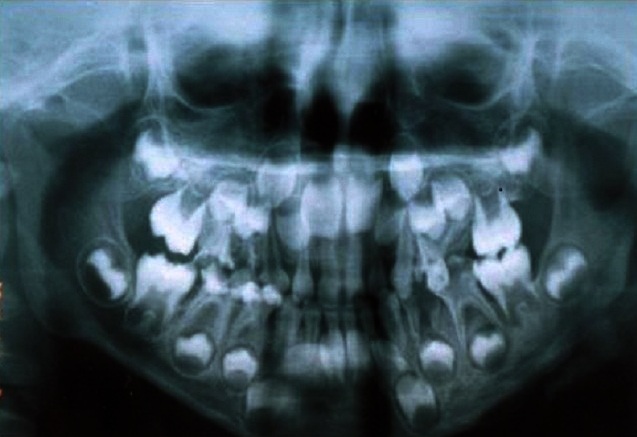
La dent 74 à l’état de racines et la 75 délabrée avec atteinte de furcation

**Figure 4:**
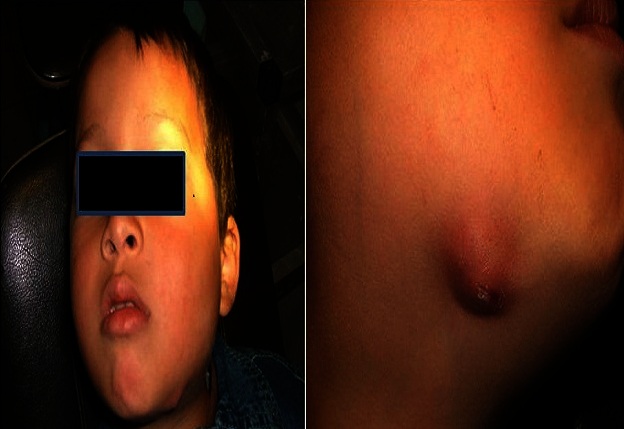
Persistance de la tuméfaction qui a évolué vers une abcédation

**Figure 5:**
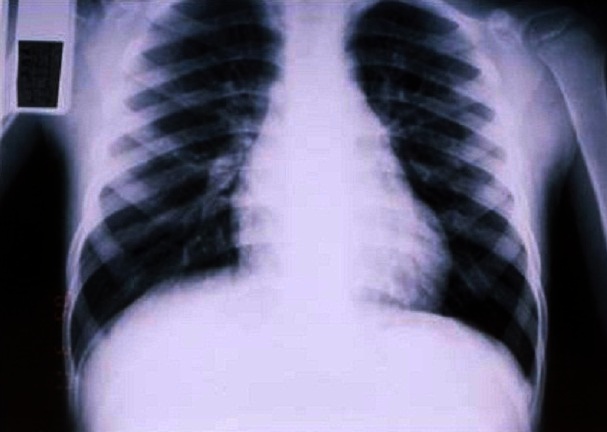
Radiographie du thorax normale

**Figure 6:**
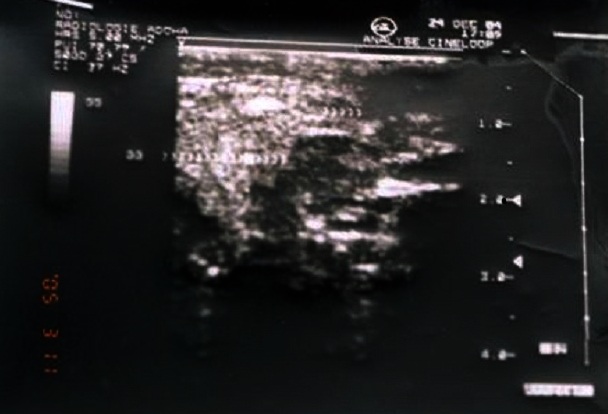
Adénopathies sous maxillaires gauches mesurant 1,8x1cm et 1,4x1cm

**Figure 7:**
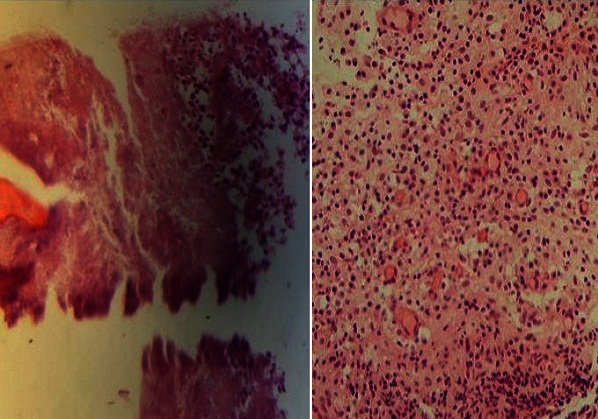
Inflammation granulomateuse polymorphe avec présence de corps d'allure actinomycosique

La radio panoramique dentaire a montré: la 74 est à l’état de racine, et la 75 avait une atteinte de la furcation (Figure 3). Le diagnostic qui a été évoqué au début est celui d'une cellulite chronique causée par l'infection des dents. Le traitement initial a consisté en l'administration d'une antibiothérapie à base d'amoxicilline ‘acide clavulanique pendant 10 jours et une corticothérapie de courte durée, avec mise en état de la cavité buccale (extraction des dents causales). Une semaine après l'extraction de la 74 et la 75, l’évolution était marquée par l'apparition d'une ulcération avec fistulisation de l'abcès, ainsi que par la persistance de l'adénopathie (Figure 4). L'enfant a été alors adressé au service d'ORL où le diagnostic de tuberculose a été suspecté. Un complément de bilan a été fait (biologique et radiographie), il a montré la présence d'adénopathie cervicale isolée (Figure 5, Figure 6). Au service d'ORL le patient a subi une incision de l'abcès avec exérèse chirurgicale de l'adénopathie et le matériel prélevé a été adressé au service d'anatomie pathologique pour examen. Ce dernier a conclu à une inflammation granulomateuse polymorphe avec Présence de corps d'allure actinomycosique (Figure 7).

## Discussion

L'actinomycose cervico-faciale a été décrite pour la première fois chez l'homme par LEHERT en 1857 [6]. C'est une infection rare, sous diagnostiquée car souvent méconnue, sa fréquence est estimée à 5/100000 habitants [7]. L'actinomycose est dix fois plus fréquente dans les communautés rurales que dans les régions urbaines. Elle prédomine dans les pays au système de santé précaire, notamment en ce qui concerne les soins dentaires [8]. La répartition selon l’âge est caractérisée par sa rareté chez l'enfant, avec seulement 20% des cas publiés [5].

L'actinomycose est une infection bactérienne, causée par un bacille anaérobie, gram positif, l'actinomyces. Il existe plusieurs types dont le plus fréquemment rencontré en pathologie humaine est *Actinomyces Israelii* Maroc. Les autres sont plus rares: A. naeslundii, A. bovis, A. viscosus, A. odontolyticus [1]. Une lésion de la muqueuse buccale s'avère nécessaire pour le développement de la maladie [3, 6]. La barrière mécanique de la muqueuse buccale est faible en deux points: le collet gingival et les cryptes linguales où l’épithélium est mince et facilement traumatisable [9]. C'est donc en ces deux points que se développe d'abord l'actinomycose. Le développement des actinomyces est favorisé essentiellement par le mauvais état bucco-dentaire, c'est le cas de notre patient. La dissémination de l'actinomycose cervico faciale se fait par contiguité et très rarement par voie hématogène [5]. Dans sa forme typique, l'actinomycose cervico-faciale passe par trois stades clinique;un stade d'infiltration qui évolue vers l'abcèdation, puis vers la fistulisation à la peau [5, 6]. Dans notre cas clinique, l’évolution s'est faite vers l'abcèdation. En effet, après l'extraction des dents, le patient n'a pas bénéficié de traitement antibiotique, ce qui a entraîné l'exacerbation de la symptomatologie avec la formation d'un abcès prêt à se fistuliser à la peau.

L'imagerie au cours de l'actinomycose est non spécifique. La radiographie panoramique n'a montré qu'un délabrement important des molaires temporaires avec atteinte de la furcation. Cependant, l’échographie a montré deux adénopathies sous maxillaires gauches. Le diagnostic positif repose sur l'examen bactériologique du pus prélevé au niveau de la lésion, qui permet de confirmer le diagnostic par la mise en évidence de bacille gram positif filamenteux. Mais ce dernier peut s'avérer délicat du fait du faible taux d'isolement de ce germe [7]. C'est l'examen anatomopathologique du matériel prélevé qui permet le plus souvent de poser le diagnostic de cette affection en identifiant le grain actinomycosique [3, 10]. Le traitement de l'actinomycose repose sur une antibiothérapie seule ou associée dans certains cas à la chirurgie [5, 7, 8, 10]. La durée du traitement antibiotique est adaptée en fonction de l’évolution clinique. Les pénicillines, cyclines, macrolides et les synergistines sont actifs sur les actinomyces [3]. En fonction de la précocité du diagnostic, le traitement peut durer de 1mois à plusieurs mois. La chirurgie est indiquée en cas de foyer infectieux abcédé fistulisé [6]. La chirurgie permettra le drainage des abcès, la mise à plat des trajets fistuleux et l'exérèse des masses nécrotiques évitant ainsi les récidives [6, 8]. La chirurgie peut être à but purement thérapeutique, où à but diagnostique et thérapeutique. Pour notre patient, le diagnostic de certitude a été posé en postopératoire car aucun diagnostic n'a pu être retenu avant l'exérèse chirurgicale. L'enfant a pu bénéficier d'une antibiothérapie adaptée par macrolides pendant un mois.

Le pronostic de l'affection dépend de la précocité du diagnostic et du traitement, car c'est une affection curable si elle est reconnue et bien traitée.

Le diagnostic différentiel histologique se pose avec les mycétomes, les nocardioses, les streptomyces, les botryomycoses, les leptothrix, l'aspergillose. L'actinomycose cervico-faciale peut évoluer sur plusieurs années s'accompagnant d'une altération progressive de l’état général, et évoluant vers le décès [10]. Concernant notre patient, le diagnostic a pu être confirmé assez rapidement, mais il a gardé quand même quelques séquelles (cicatrices dysesthésique). L'hygiène buccodentaire est la clé de la prévention contre les actinomycoses cervico-faciales, puisque c'est le point de départ de cette affection [5]. La prévention passe par le traitement et l’éradication de tous les foyers infectieux bucco-dentaires, pouvant constituer un abri pour l'actinomyces. Un traitement des infections de la sphère ORL est également nécessaire [8].

## Conclusion

L'actinomycose cervico-faciale, est une pathologie grave et potentiellement mortelle. Elle doit être présente à l'esprit, car reconnue précocement et traitée correctement, sa guérison est habituelle.
